# A nationwide study of the long-term prevalence of dementia and its risk factors in the Swedish intensive care cohort

**DOI:** 10.1186/s13054-020-03203-y

**Published:** 2020-09-04

**Authors:** Björn Ahlström, Ing-Marie Larsson, Gunnar Strandberg, Miklos Lipcsey

**Affiliations:** 1 Department of Surgical Sciences, Uppsala University, Uppsala, Sweden; 2 Region Dalarna, Centre of Clinical Research Dalarna, Nissers väg 3, Falu lasarett, Falun, 79182 Sweden; 3 Hedenstierna Laboratory, CIRRUS, Anesthesiology and Intensive Care, Department of Surgical Sciences, Uppsala University, Uppsala, Sweden

**Keywords:** Critical care, Sepsis, Dementia, Risk factors, Cohort studies

## Abstract

**Background:**

Developing dementia is feared by many for its detrimental effects on cognition and independence. Experimental and clinical evidence suggests that sepsis is a risk factor for the later development of dementia. We aimed to investigate whether intensive care-treated sepsis is an independent risk factor for a later diagnosis of dementia in a large cohort of intensive care unit (ICU) patients.

**Methods:**

We identified adult patients admitted to an ICU in 2005 to 2015 and who survived without a dementia diagnosis 1 year after intensive care admission using the Swedish Intensive Care Registry, collecting data from all Swedish general ICUs. Comorbidity, the diagnosis of dementia and mortality, was retrieved from the Swedish National Patient Registry, the Swedish Dementia Registry, and the Cause of Death Registry. Sepsis during intensive care served as a covariate in an extended Cox model together with age, sex, and variables describing comorbidities and acute disease severity.

**Results:**

One year after ICU admission 210,334 patients were alive and without a diagnosis of dementia; of these, 16,115 (7.7%) had a diagnosis of sepsis during intensive care. The median age of the cohort was 61 years (interquartile range, IQR 43–72). The patients were followed for up to 11 years (median 3.9 years, IQR 1.7–6.6). During the follow-up, 6312 (3%) patients were diagnosed with dementia. Dementia was more common in individuals diagnosed with sepsis during their ICU stay (log-rank *p* < 0.001), however diagnosis of sepsis during critical care was not an independent risk factor for a later dementia diagnosis in an extended Cox model: hazard ratio (HR) 1.01 (95% confidence interval 0.91–1.11, *p* = 0.873). Renal replacement therapy and ventilator therapy during the ICU stay were protective. High age was a strong risk factor for later dementia, as was increasing severity of acute illness, although to a lesser extent. However, the severity of comorbidities and the length of ICU and hospital stay were not independent risk factors in the model.

**Conclusion:**

Although dementia is more common among patients treated with sepsis in the ICU, sepsis was not an independent risk factor for later dementia in the Swedish national critical care cohort.

**Trial registration:**

This study was registered a priori with the Australian and New Zeeland Clinical Trials Registry (registration no. ACTRN12618000533291).

## Background

Dementia is a common and often detrimental group of diseases with a sharply increasing incidence in the elderly [1, 2]. In general, the disease is characterized by memory loss, disturbances in language, altered perception, and other psychological and psychiatric changes. Together, these symptoms cause impaired daily functioning [3] and may severely affect health-related quality of life [4].

In hospitalized patients, and especially in the intensive care population, sepsis is a common syndrome [5] defined by a dysregulated host response to an infection [6]. The systemic inflammation in sepsis has been suggested to have a long-term negative impact on the brain [7]. Both short- and long-term effects of experimental sepsis on brain cells and behavior have been described in rodents [8–10]. Septic encephalopathy and persisting cognitive disturbances have also been reported in human studies [11–13]. Moreover, in patients with an assessment of cognitive function before and after hospitalization, a decline in cognitive function was more pronounced after hospitalization for severe sepsis than after admission for other reasons [14]. Sepsis diagnosis has also been reported to be more common in the history of patients with dementia than age- and sex-matched controls from the health care system [15]. Finally, sepsis is an independent risk factor for dementia in observational studies [16, 17]. Based on these data, we hypothesized that dementia would develop more commonly in patients admitted for, or developing, sepsis in intensive care compared with other patient groups.

However, the prevalence of dementia in the population is relatively low [1]. Additionally, dementia is usually a slowly developing syndrome with a long subclinical period before diagnosis [18]. Dementia also increases the risk of sepsis [19], and several comorbidities are risk factors for both dementia and sepsis.

We therefore set out to investigate our hypothesis in a large nationwide database with an extended follow-up and accounting for comorbidities.

Our primary endpoint was the hazard ratio (HR) of sepsis for a diagnosis of dementia adjusting for several potential risk factors. We also investigated the impact of these risk factors and crude dementia incidence in this cohort.

## Methods

This study was approved by the Regional Ethics Committee of Uppsala (approval no. 2016/421). Since this is a registry-based study, informed consent was waived. The protocol of the study was registered a priori with the Australian and New Zeeland Clinical Trials Registry (registration no. ACTRN12618000533291). Reporting strictly follows the STROBE Statement [20].

### Data sources

The Swedish Intensive Care Registry (SIR) is a national registry to which all general ICUs in Sweden are reporting data on all admissions [21, 22]. The National Patient Register (NPR) includes data from all in-patient hospital visits in Sweden, and the Cause of Death Registry includes deaths of all Swedish residents, including all deaths abroad [23]. Both registries are run by the Swedish National Board of Health and Welfare. The Swedish Dementia Registry (SveDem) is a national quality registry started in 2007, with primary care and specialized memory units reporting cases in Sweden. The SveDem had an estimated coverage ratio of 27–35% of dementia incidence in Sweden in 2017, partly overlapping with the NPR [24, 25].

### Cohorts

All adult patients aged > 17 years who had at least one episode of intensive care in the SIR in 2005 to 2016 were included. We excluded patients with a diagnosis of dementia at ICU admission and patients who died or acquired a diagnosis of dementia during the first year after ICU admission.

Patients in the Sepsis diagnosis code cohort (henceforth referred to as the Sepsis cohort) were identified by sepsis diagnosis codes registered in the SIR. The diagnosis of severe sepsis and septic shock, represented as ICD-10 A41.9 (2005–2010), R65.1 or R57.9 (2011–2016), has to be confirmed or negated when registering a patient in the SIR. Those without sepsis diagnosis codes in SIR were included in the No sepsis diagnosis code cohort (hereafter referred to as the No Sepsis cohort). During the entire study period, the SIR defined the diagnosis of sepsis, severe sepsis, and septic shock according to the second International Sepsis Definitions Conference of 2001 [26].

### Data

From the SIR, we extracted data on the severity of illness at admission, invasive ventilator support, renal replacement therapy (RRT), ICU length of stay (ICU-LoS), and diagnoses relevant to the ICU episode. For patients with repeated admissions, we used the first ICU episode with a sepsis diagnosis code or, for patients without a sepsis diagnosis code, the first ICU episode in the SIR. We treated overlapping ICU episodes as one episode. The severity of illness was initially reported as Acute Physiology, Age, Chronic Health Evaluation II (APACHE II) in the SIR [27] and, during 2010, gradually substituted with the Simplified Acute Physiology Score 3 (SAPS3) [28].

Death date was extracted from the Cause of Death Registry. From the NPR, we derived ICD-10 [29] diagnosis codes for all inpatient care episodes from 5 years before the ICU care episode to December 31, 2016. The revised Charlson Comorbidity Index (CCI) [30] was calculated using diagnoses from all health care contacts preceding or coinciding with the first ICU episode.

We defined dementia using the following ICD-10 codes according to the CCI [31]: F00x-F03x, F051, and G30x-31x. Because the Inpatient Care Diagnoses database only includes diagnoses from inpatient care, we incorporated data from the SveDem to track down patients with dementia not admitted to the hospital. The date of dementia diagnosis was the first occurrence of the condition in the NPR or the SveDem.

The Swedish Dementia Centre, commissioned by the Swedish National Board of Health and Welfare, provides recommendations on the diagnostic process in suspected dementia. In both primary and specialist care, the recommendation is to use the diagnostic criteria of the ICD-10, especially identifying the importance of symptom stability (6 months) and the exclusion of co-existing confusion [32].

### Statistics

For descriptive statistics, we used counts with percentages, means with standard deviations and medians and interquartile ranges (IQR) as appropriate.

We assessed the crude incidence of dementia with Kaplan-Meier curves using the log-rank test. For the primary analysis, HRs for the risk of dementia were calculated in a Cox regression model with mortality censored. The following covariates were chosen from available variables through directed acyclic graph analysis and a literature review: sepsis; age [1] and sex, all of which have been previously described as independent risk factors for dementia [33]; CCI; SAPS3 box 2+3; hospital length of stay (H-LoS); ICU-LoS; invasive ventilator therapy; and RRT. Missing data were substituted by redundancy between data sources where possible. Missing SAPS3 box 2+3 was substituted by multiple imputations into five datasets using the Multivariate Imputation by Chained Equations (MICE) package in R [34]. The results from the analyses on the imputation datasets were pooled using the Harrel Miscellaneous (Hmisc) package in R.

The proportional hazards assumption was deemed fulfilled after visual inspection of plots of scaled Schoenfeld residuals against time and the covariates treated as continuous were evaluated for linearity by plots of Martingale residuals against the covariate. Because of nonlinearity for all continuous covariates, we used cubic splines in the Cox model [35]. Seven sensitivity analyses were performed according to the description in Additional file 1.

We defined statistical significance as *p* < 0.05. HRs for which cubic splines were applied were calculated between the 25th and 75th percentiles. Data management and descriptive statistics were performed in SPSS for Windows version 24 (Microsoft Inc., IL, USA). For inference tests (i.e., regression analyses) and multiple imputations, we used R Software version 3.5.3 (The R Foundation for Statistical Computing, Vienna, Austria; https://www.r-project.org).

## Results

**Fig. 1:**
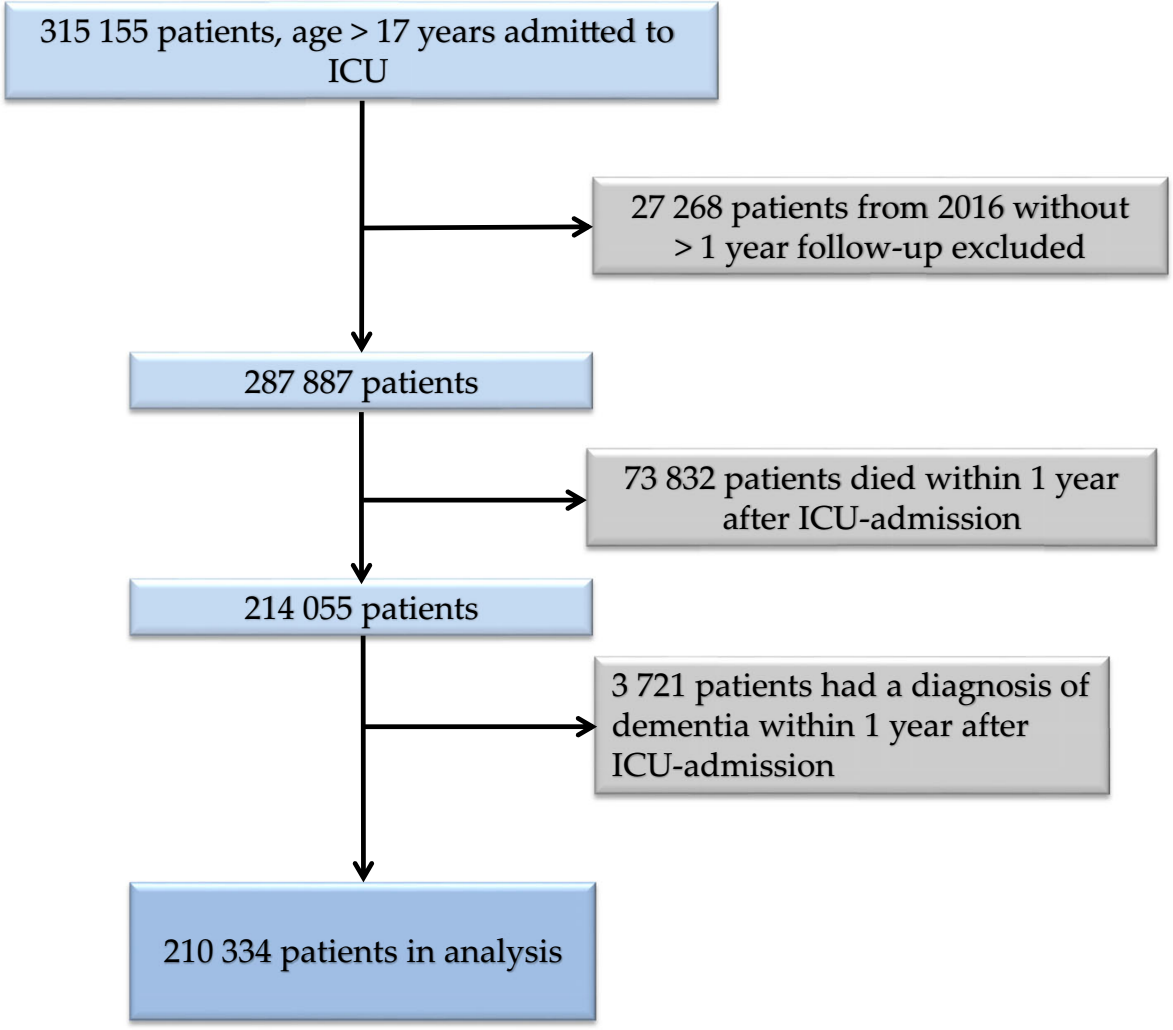
Patient selection flowchart. ICU, intensive care unit. Template adopted from the PRISMA-statement [39]

Of 315,155 patients, 210,334 (67%) were still alive and without dementia 1 year after ICU admission (Fig. 1). Of those 210,334 patients, 16,115 (8%) had a sepsis diagnosis code in ICU care. The patients were followed for a median of 3.9 years (IQR 1.7–6.6). SAPS3 data were completely missing in 23.8% of the patients in the Sepsis cohort and 45.2% in the No sepsis cohort. Of patients with missing SAPS3, 46% had a registration of an APACHE II score in the Sepsis cohort and 23% in the No sepsis cohort. Missing SAPS3 data were imputed. Of all patients admitted to the ICU in 2005–2016, 8495 (4.0%) emigrated at least once from Sweden, and of these, 5358 (63.1%) had at least one listing in the NPR or the SveDem > 1 year after ICU admission.

Patients in the Sepsis cohort were older, had higher SAPS3, and had more comorbidities, expressed as a higher CCI score. They also had longer ICU-LoS and H-LoS and were more commonly treated with invasive ventilation and RRT (Table 1). During follow-up, the Sepsis cohort had a mean of 3.9 episodes of inpatient care, whereas 32% of these patients had no such episode. The No sepsis cohort had a mean of 3.4 episodes of inpatient care, and 38% of these patients were without any such episode.

**Table 1:** Characteristics of adult patients treated in Swedish ICUs, from 2005 to 2015 1 year after ICU admission

	Sepsis diagnosis code	No sepsis diagnosis code	All
Number of patients	16,115 (7.7)	194,219 (92.3)	210,334 (100)
Female sex	6954 (43.2)	79,803 (41.1)	86,757 (41.2)
Age at ICU admission (years)	66 (54–74)	61 (42–72)	61 (43–72)
Hospital type			
University	4304 (26.7)	81,560 (42.0)	85,864 (40.8)
County	7989 (49.6)	73,442 (37.8)	81,431 (38.7)
District	3822 (23.7)	39,217 (20.2)	43,039 (20.5)
SAPS3	61 (53–70)	45 (37–55)	47 (38–57)
CCI score	1 (0–2)	0 (0–2)	0 (0–2)
Hospital LoS (days)	20 (11–41)	11 (5–21)	11 (5–22)
ICU-LoS (days)	2.94 (1.27–7.70)	0.91 (0.55–1.89)	0.94 (0.58–2.06)
RRT	1492 (9.3)	2019 (1.0)	3511 (1.7)
RRT time (minutes)	6330 (3506–11,672)	3796 (1800–8280)	4560 (2370–9695)
Ventilator	4897 (30.4)	40,221 (20.7)	45,118 (21.5)
Ventilator time (minutes)	6240 (2370–14,160)	540 (180–2245)	720 (190–3150)
Surgery	2448 (15.2)	34,229 (17.6)	36,677 (17.5)
Surgery, acute	2048 (12.7)	19,109 (9.8)	21,157 (10.1)

Data are presented as numbers with percentages or medians with interquartile ranges as appropriate

*ICU* intensive care unit, *LoS* Length of stay, *SAPS3* Simplified Acute Physiology Score 3, *CCI* revised Charlson Comorbidity Index, *RRT* renal replacement therapy

The 6312 patients ultimately developing dementia were older and predominantly female and had higher SAPS3 and longer ICU-LoS and H-LoS than those without dementia (Table 2). In addition, those patients who developed dementia were less frequently treated with RRT and invasive ventilator therapy. Finally, patients with dementia had a higher rate of acute surgical admissions, but a lower rate of planned surgical admissions. The size of these differences was generally small, however.

**Table 2:** Characteristics of patients alive without dementia 1 year after ICU admission stratified on receiving dementia diagnosis later

	Dementia diagnosis code	No dementia diagnosis code	All
Number of patients	6312 (3)	204,022 (97)	210,334
Sepsis	472 (7.5)	156,439 (7.7)	16,115 (7.7)
Female sex	2785 (44.1)	83,973 (41.2)	86,758 (41.2)
Age at ICU admission (years)	76 (70–81)	60 (43–71)	61 (43–72)
Hospital type			
University	2448 (38.8)	83,416 (40.9)	85,864 (40.8)
County	2447 (38.8)	78,984 (38.7)	81,431 (38.7)
District	1417 (22.4)	41,622 (20.4)	43,039 (20.5)
SAPS3	55 (48–63)	47 (38–57)	47 (38–57)
CCI score	0 (0–2)	0 (0–2)	0 (0–2)
ICU LoS (days)	0.98 (0.68–2.2)	0.94 (0.58–2.1)	0.94 (0.58–2.1)
Hospital LoS (days)	15 (9–26)	11 (5–22)	11 (5–22)
RRT	57 (0.9)	3454 (1.7)	3511 (1.7)
RRT (minutes)	4670 (2515–9487)	4560 (2370–9697)	4560 (2370–9695)
Ventilator	990 (15.7)	44,128 (21.6)	45,118 (21.5)
Ventilator time (minutes)	705 (218–3331)	720 (190–3150)	720 (190–3153)
Surgery	1087 (17.3)	35,580 (17.5)	36,677 (17.5)
Surgery, acute	642 (10.2)	20,515 (10.1)	21,157 (10.1)

Data are presented as numbers with percentages or medians with interquartile ranges as appropriate

*ICU* intensive care unit, *LoS* Length of stay, *SAPS3* Simplified Acute Physiology Score 3, *CCI* revised Charlson Comorbidity Index, *RRT* renal replacement therapy

**Fig. 2:**
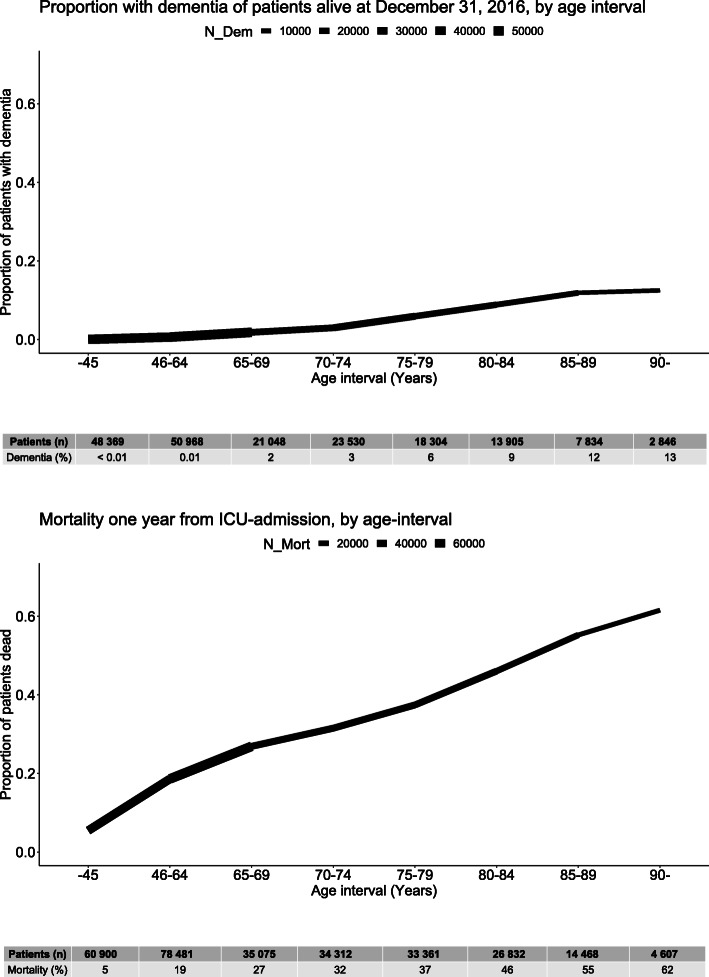
Dementia prevalence by age interval in patients of the ICU cohort alive at 31st of December 2016 and mortality 1 year from ICU admission. Line thickness represents the number of patients at risk. ICU, intensive care unit; N_Mort, number at risk of mortality; N_Dem, number at risk of dementia

**Fig. 3:**
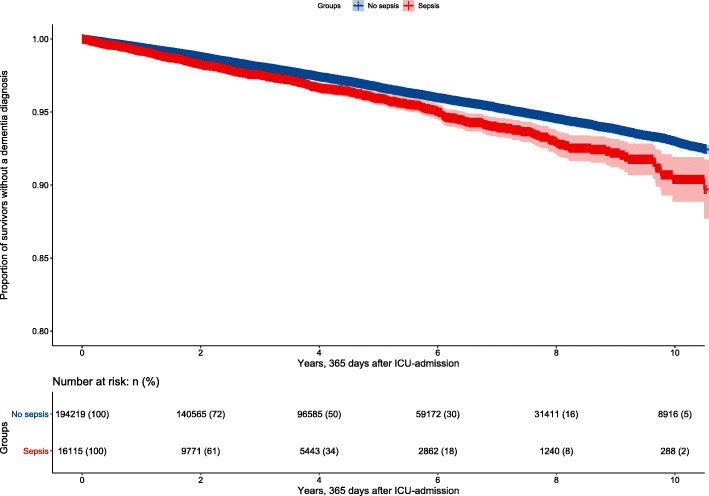
Kaplan-Meier curves (95% CI) for dementia showing patients with No sepsis diagnosis code (No-sepsis) and with Sepsis diagnosis code (Sepsis) initially having survived without dementia for 1 year after ICU admission. Log-Rank *P* < 0.001. CI, confidence interval ICU intensive care unit

**Fig. 4:**
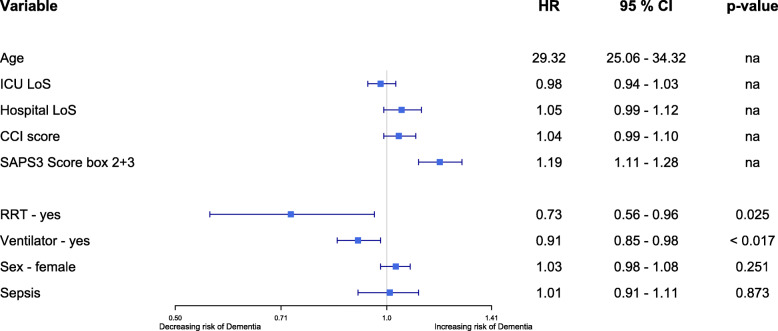
Forest-plot of Cox-regression model for dementia > 1 year after ICU admission for patients > 17 years treated in Swedish ICUs during the years 2005 to 2015. For variables treated as continuous: age, ICU-LoS, hospital LoS, CCI score, and SAPS3 box 2+3, the HR is of the difference between the 25th and 75th percentiles. HR, hazard ratio; CI, confidence interval; ICU, intensive care unit; LoS, length of stay; CCI, revised Charlson Comorbidity Index; SAPS3, Simplified Acute Physiology Score 3; RRT, renal replacement therapy

Dementia prevalence and 1-year mortality increased with age in patients admitted to the ICU during the study and alive on the last day of follow-up (December 31, 2016) (Fig. 2). In the unadjusted analysis, dementia was more common in individuals diagnosed with sepsis during their ICU stay (log-rank *p* < 0.001) as depicted in the Kaplan-Meier survival curve (Fig. 3). However, after adjusting for age, sex, CCI score, SAPS3 box 2+3, H-LoS, ICU-LoS, invasive ventilator therapy, and RRT, sepsis was no longer an independent risk factor for dementia (HR 1.01, 95% CI 0.91–1.11) (Fig. 4). Only age, SAPS3 box 2+3, RRT, and ventilator therapy were independent risk factors in the model, with the HR for dementia increasing with increasing age and SAPS3 box 2+3 but decreasing with the use of RRT and ventilator therapy.

### Sensitivity analyses

In any of the pre-specified sensitivity analyses, sepsis was not an independent risk factor for dementia (Additional file 2).

## Discussion

In our nationwide Swedish cohort of 210,334 patients alive without dementia 1 year after ICU admission, sepsis was found to be a crude risk factor for a later diagnosis of dementia. However, after adjusting for baseline characteristics of the patients in our cohort, sepsis was no longer an independent risk factor for dementia. This finding was consistent in all performed sensitivity analyses.

In a previous study [36], 516 patients having survived a sepsis episode were compared with 4517 patients having survived a hospitalization without sepsis. All included individuals underwent at least one prospective cognitive testing. When followed for up to 1 year, the sepsis patients performed worse on repeated cognitive testing. However, it is not clear whether this condition evolves into fulfilling the diagnostic criteria of dementia, and the authors did not control for age, comorbidities, or the degree of acute illness. In the present study, we controlled for several factors expressing both comorbidity and the degree of acute illness besides age. Guerra et al. performed two studies on a 2005 Medicare cohort. In their first study [16], the findings seem to confirm the hypothesis that the higher rate of observed cognitive dysfunction after sepsis is evolving into a higher rate of clinically diagnosed dementia in patients treated in the ICU with sepsis than in those treated in the ICU without sepsis during their hospitalization. However, they were only able to adjust for comorbidities diagnosed in the year preceding the index hospitalization, thereby risking underestimating the comorbidities. In addition, the authors run the risk of overlooking the presence of dementia diagnoses in earlier years that was not registered in the year preceding hospital admission. Furthermore, because dementia is a syndrome of a slowly evolving disease [18], dementia diagnosed early after the index hospitalization might be an example of a clinically overt disease coming to the attention of the medical system in the convalescence period after hospitalization rather than a consequence of the sepsis episode or acute illness per se. Dementia may also be a risk factor for sepsis [19]. We sought to lessen the effect of both over-diagnosis due to hospitalization and causality problems between sepsis and dementia by excluding dementia diagnoses registered during the first year after ICU admission. In the second study by Guerra et al. [17], patients admitted to the ICU with sepsis were compared with non-hospitalized patients matched on age group, sex, and race. Sepsis was a significant risk factor for dementia. However, in a model adjusting for comorbid diagnoses associated with dementia during the index hospitalization, the effect of sepsis decreased compared with using the same comorbidities diagnosed before the index hospitalization. Moreover, when using comorbidities diagnosed during the index quarter in the model, the effect of sepsis on the risk of dementia disappeared in line with our findings. In a case-control study [15], the odds ratio for having had a sepsis diagnosis in 5955 patients with a dementia diagnosis was higher than in age- and sex-matched controls without dementia. In their design, the authors did not account for the amount of time elapsed from sepsis to the diagnosis of dementia, nor did they adjust for comorbidities diagnosed during the index hospitalization.

Despite that the patients in the Sepsis and the No sepsis cohorts are from the same ICU cohort, they were not comparable, i.e., sepsis cohort patients had more chronic illnesses, were older, and had a higher degree of acute illness. Hence, it was essential to adjust to these specific factors. Our study used the revised CCI as a composite measure of the comorbid burden of each patient. We also adjusted for the severity of acute illness, as we presumed that it might mediate the effect of sepsis on dementia development. SAPS3 box 2+3, i.e., acute illness severity, was an independent risk factor for dementia. However, in a sensitivity analysis in which SAPS3 box 2+3 was excluded from the model, sepsis was not a significant risk factor for dementia. This finding implies that SAPS3 box 2+3 does not modulate the effect of sepsis in the model.

As expected, age was a strong risk factor for developing dementia during follow-up in our cohort of ICU-treated patients. Intriguingly, in the oldest age category (> 90 years), we observed a lower prevalence of dementia compared with another study on a Swedish cohort [33]. This lower prevalence may be related, in part, to the very high mortality rates in this elderly patient group that have been treated in the ICU. Surprisingly, the CCI score was not an independent risk factor for dementia in the model. We speculate that this finding is due to the small difference between the 25th and 75th percentiles of the CCI score. A wider range of CCI might have yielded another result. For the variables in which cubic splines were applied, the HR was calculated for the difference between values at the 25th and 75th percentiles to reduce the risk of false conclusions from the HRs of these splined variables. Still, despite this adjustment, results for the splined variables need to be interpreted cautiously. However, their validity as covariates of sepsis—the primary endpoint of the study—in the model is higher after cubic spline application than if we would have categorized these nonlinear variables [37].

### Strengths and limitations

A major strength of this study is that our sample contains virtually all intensive care patients in Sweden over 11 years (2005 to 2015), which represents the different socioeconomic groups of a high-income country. Another strength is the possibility to follow the patients for an extended period before ICU admission. Such an approach allows for an accurate assessment of comorbidities in general and pre ICU dementia in particular. We believe that the long mean follow-up of > 4 years from 1-year post-ICU admission is also an important asset of the study in that most types of dementia are gradually developing diseases [38]. Our study also has by far the largest cohort of ICU survivors to study the association between sepsis and dementia. Finally, we were able to control for the severity of acute illness.

Our study has some limitations of note. The major limitation is that we define dementia as a dementia diagnosis at an inpatient visit at a hospital or a dementia diagnosis in the SveDem. We thus expect to miss a proportion of patients despite using data from several registries to detect dementia in both hospital-admitted patients and outpatients. However, we have no reason to believe that dementia would be unreported to a larger extent in patients treated for sepsis in the ICU as the Sepsis cohort patients had more hospital inpatient visits than the No sepsis cohort patients during follow-up. This observation is possibly due to the higher comorbidity burden and higher age in the Sepsis cohort patients. Moreover, we choose not to include patients that did not live up to 1 year after ICU admission, resulting in excluding almost one third of the patients in the analysis. However, we found in a sensitivity analysis that our results did not change after including those patients not surviving up to 1 year after ICU admission. Another potential shortcoming of the data is the possibility the patients die before they develop dementia, implying that the findings of this study could potentially need to be verified in cohorts with much lower mortality. In a sensitivity analysis, we included only patients with SAPS3 in the lowest quartile expecting a lower mortality and thus a smaller risk of dying acting as a competing event, but sepsis was not an independent risk factor in this analysis. Finally, the present study is limited because 4% of the patients emigrated from Sweden at some point during the study. However, 63% of these patients had at least one listing in our data sources during the follow-up, which also were the case in the complete cohort. Thus, we chose not to exclude emigrated patients from the main analysis; however, doing so in a sensitivity analysis did not affect our results.

### Further research

Because the effect of sepsis on the risk of later dementia development has been shown to be minimal in previous studies and not present at all in our study, we recommend that further research on outcome after sepsis be directed in other directions.

## Conclusion

In conclusion, although dementia was more common in the whole nation Swedish ICU cohort for 2005–2015 treatment for sepsis in the ICU, sepsis was not a risk factor for later dementia after adjustment for pre-specified, relevant, baseline variables. In our sample, acute illness severity altered the risk of dementia, which might account for a fraction of the apparent causality between sepsis and dementia in ICU patients.

## Supplementary information

**Additional file 1.** Listing of performed sensitivity analyses and their rationale.

**Additional file 2.** Results of performed sensitivity analyses.

## Data Availability

The data used in this study are available from the SIR, the NPR, and the SveDem. However, privacy or ethical restrictions apply to the availability of these data, which were used under license for the current study. Thus, these data are not publicly available. The data, however, are available from the authors upon reasonable request and with permission of the SIR, the NPR, and the SveDem.

## Abbreviations

APACHE II: Acute Physiology, Age, Chronic Health Evaluation II
CCI: Revised Charlson Comorbidity Index
H-LoS: Hospital length of stay
HR: Hazard ratio
ICD-10: International Classification of Diseases, Tenth Revision
ICU: Intensive care unit
ICU-LoS: Intensive care unit length of stay
LoS: Length of stay
MICE: Multivariate Imputation by Chained Equations
NPR: National Patient Registry
RRT: Renal replacement therapy
SAPS3: Simplified Acute Physiology Score 3
SIR: Swedish Intensive Care Registry
SveDem: Swedish Dementia Registry
STROBE: Strengthening the Reporting of Observational Studies in Epidemiology
