# Supplementary material for: A nationwide study of the long-term prevalence of dementia and its risk factors in the Swedish intensive care cohort
Source: Crit Care. 2020 Sep 4;24:548. doi: 10.1186/s13054-020-03203-y (PMC7472680; doi:10.1186/s13054-020-03203-y)
Supplement: Supplementary file 1 — Additional file 1. Listing of performed sensitivity analyses and their rationale. [file 13054_2020_3203_MOESM1_ESM.docx]

Additional file 1. Listing of performed sensitivity analyses and their rationale.

|  |  | Sensitivity analysis |  | Rationale |
| --- | --- | --- | --- | --- |
|  |  |  |  |  |
| 1 |  | Without imputed SAPS3 data |  | to ensure that, the imputation did not skew the results |
|  |  |  |  |  |
| 2 |  | With dementia diagnoses from ICU discharge to three years post ICU in patients 65 years or older |  | in order to explore the effect of dementia diagnoses early after the ICU-care episode |
|  |  |  |  |  |
| 3 |  | Using dementia diagnosis from ICU stay |  | in order to find the possible effect of our one-year buffer period from ICU admission; |
|  |  |  |  |  |
| 4 |  | Comparing individuals with septic shock to individuals without any sepsis diagnosis during the years 2011-2016 |  | to find a possible dose-response effect in sepsis severity |
|  |  |  |  |  |
| 5 |  | Excluding individuals with the known risk factors for dementia: depression, surgery on extracorporeal circulation, diabetes, neurotrauma, minor cognitive deficit, and stroke in the five years preceding ICU-admission |  | to explore the possible effect och specific risk factors of dementia |
|  |  |  |  |  |
| 6 |  | Excluding SAPS3 box 2 + 3 from the model |  | to explore the effect of SAPS3 box 2+3 on spesis in the model |
|  |  |  |  |  |
| 7 |  | Excluding emigrated patients |  | to ensure that the possible loss to follow-up did not skew the results |
|  |  |  |  |  |
|  |  |  |  |  |
| 8 |  | Only including patients with SAPS3 in the 1st quartile (SAPS3 = 24 - 26 points) |  | to lessen the impact of death as a competing risk on the risk of dementia |
|  |  |  |  |  |
|  |  | ICU: Intensive Care Unit |  |  |
|  |  | SAPS3: the Simplified Acute Physiology Score 3 |  |  |
