# Supplementary material for: A nationwide study of the long-term prevalence of dementia and its risk factors in the Swedish intensive care cohort
Source: Crit Care. 2020 Sep 4;24:548. doi: 10.1186/s13054-020-03203-y (PMC7472680; doi:10.1186/s13054-020-03203-y)
Supplement: Supplementary file 2 — Additional file 2. Results of performed sensitivity analyses. [file 13054_2020_3203_MOESM2_ESM.docx]

Additional file 2. Results of performed sensitivity analyses

| Sensitivity analysis no |  | Variable |  | Effect (HR) |  | Lower 95 % CI limit |  | Upper 95 % CI limit |
| --- | --- | --- | --- | --- | --- | --- | --- | --- |
|  |  |  |  |  |  |  |  |  |
| 1 |  | **Without imputed SAPS3 data** | | | |  |  |  |
|  |  | Age |  | 32.65 |  | 25.05 |  | 42.55 |
|  |  | ICU LoS |  | 0.89 |  | 0.83 |  | 0.95 |
|  |  | Hospital LoS |  | 1.21 |  | 1.10 |  | 1.32 |
|  |  | CCI score |  | 1.03 |  | 0.94 |  | 1.11 |
|  |  | SAPS3 Box 2+3 |  | 1.31 |  | 1.17 |  | 1.46 |
|  |  | RRT - Yes |  | 0.67 |  | 0.49 |  | 0.92 |
|  |  | Ventilator - Yes |  | 0.80 |  | 0.72 |  | 0.89 |
|  |  | Sex - Female |  | 1.01 |  | 0.93 |  | 1.09 |
|  |  | Sepsis - Yes |  | 0.90 |  | 0.79 |  | 1.02 |
|  |  |  |  |  |  |  |  |  |
| 2 |  | **With dementia diagnoses from ICU discharge to three years post ICU in patients 65 years or older** | | | | | | |
|  |  | Age |  | 0.00 |  | 0.00 |  | 475.52 |
|  |  | ICU LoS |  | 0.93 |  | 0.88 |  | 0.98 |
|  |  | Hospital LoS |  | 1.12 |  | 1.03 |  | 1.22 |
|  |  | CCI score |  | 0.98 |  | 0.92 |  | 1.05 |
|  |  | SAPS3 Box 2+3 |  | 1.23 |  | 1.10 |  | 1.37 |
|  |  | RRT - Yes |  | 0.66 |  | 0.47 |  | 0.91 |
|  |  | Ventilator - Yes |  | 0.79 |  | 0.73 |  | 0.87 |
|  |  | Sex - Female |  | 1.02 |  | 0.95 |  | 1.09 |
|  |  | Sepsis - Yes |  | 1.02 |  | 0.91 |  | 1.14 |
|  |  |  |  |  |  |  |  |  |
| 3 |  | **Using dementia diagnoses from ICU stay** | | | | | | |
|  |  | Age |  | 27.22 |  | 23.62 |  | 31.37 |
|  |  | ICU LoS |  | 0.96 |  | 0.93 |  | 1.00 |
|  |  | Hospital LoS |  | 1.10 |  | 1.05 |  | 1.16 |
|  |  | CCI score |  | 1.01 |  | 0.96 |  | 1.06 |
|  |  | SAPS3 Box 2+3 |  | 1.22 |  | 1.14 |  | 1.30 |
|  |  | RRT - Yes |  | 0.73 |  | 0.58 |  | 0.91 |
|  |  | Ventilator - Yes |  | 0.87 |  | 0.82 |  | 0.93 |
|  |  | Sex - Female |  | 1.02 |  | 0.97 |  | 1.06 |
|  |  | Sepsis - Yes |  | 0.99 |  | 0.91 |  | 1.08 |
|  |  |  |  |  |  |  |  |  |
| 4 |  | **Comparing individuals with septic shock with individuals without any sepsis diagnosis during the years 2011-2016** | | | | | | |
|  |  | Age |  | 37.52 |  | 27.64 |  | 50.93 |
|  |  | ICU LoS |  | 0.99 |  | 0.92 |  | 1.07 |
|  |  | Hospital LoS |  | 1.04 |  | 0.94 |  | 1.14 |
|  |  | CCI score |  | 1.02 |  | 0.93 |  | 1.11 |
|  |  | SAPS3 Box 2+3 |  | 1.22 |  | 1.08 |  | 1.37 |
|  |  | RRT - Yes |  | 0.71 |  | 0.49 |  | 1.01 |
|  |  | Ventilator - Yes |  | 0.76 |  | 0.69 |  | 0.84 |
|  |  | Sex - Female |  | 1.03 |  | 0.94 |  | 1.11 |
|  |  | Sepsis - Yes |  | 0.95 |  | 0.75 |  | 1.20 |
|  |  |  |  |  |  |  |  |  |
| 5 |  | **Excluding individuals with known risk factors for dementia: depression, surgery on extracorporeal circulation, diabetes neurotrauma, minor cognitive deficit, and stroke in the five years preceding ICU-admission** | | | | | | |
|  |  | Age |  | 38.37 |  | 29.43 |  | 50.03 |
|  |  | ICU LoS |  | 0.95 |  | 0.88 |  | 1.02 |
|  |  | Hospital LoS |  | 1.12 |  | 1.02 |  | 1.24 |
|  |  | CCI score |  | 0.94 |  | 0.86 |  | 1.04 |
|  |  | SAPS3 Box 2+3 |  | 1.11 |  | 0.95 |  | 1.31 |
|  |  | RRT - Yes |  | 0.66 |  | 0.41 |  | 1.07 |
|  |  | Ventilator - Yes |  | 0.88 |  | 0.77 |  | 1.02 |
|  |  | Sex - Female |  | 1.00 |  | 0.92 |  | 1.09 |
|  |  | Sepsis - Yes |  | 1.00 |  | 0.87 |  | 1.16 |
|  |  |  |  |  |  |  |  |  |
| 6 |  | **Excluding SAPS3 box 2 + 3 from the model** | | | | | | |
|  |  | Age |  | 29.29 |  | 25.03 |  | 34.27 |
|  |  | ICU LoS |  | 0.99 |  | 0.95 |  | 1.03 |
|  |  | Hospital LoS |  | 1.06 |  | 1.00 |  | 1.12 |
|  |  | CCI score |  | 1.06 |  | 1.00 |  | 1.11 |
|  |  | RRT - Yes |  | 0.75 |  | 0.58 |  | 0.99 |
|  |  | Ventilator - Yes |  | 0.95 |  | 0.88 |  | 1.02 |
|  |  | Sex - Female |  | 1.03 |  | 0.98 |  | 1.08 |
|  |  | Sepsis - Yes |  | 1.07 |  | 0.97 |  | 1.17 |
|  |  |  |  |  |  |  |  |  |
| 7 |  | **Excluding emigrated patients** | | | | | | |
|  |  | Age |  | 29.56 |  | 25.15 |  | 34.74 |
|  |  | ICU LoS |  | 0.98 |  | 0.93 |  | 1.02 |
|  |  | Hospital LoS |  | 1.05 |  | 0.99 |  | 1.12 |
|  |  | CCI score |  | 1.04 |  | 0.99 |  | 1.10 |
|  |  | SAPS3 Box 2+3 |  | 1.19 |  | 1.11 |  | 1.28 |
|  |  | RRT - Yes |  | 0.73 |  | 0.56 |  | 0.96 |
|  |  | Ventilator - Yes |  | 0.92 |  | 0.85 |  | 0.99 |
|  |  | Sex - Female |  | 1.03 |  | 0.98 |  | 1.08 |
|  |  | Sepsis - Yes |  | 1.00 |  | 0.90 |  | 1.11 |
|  |  |  |  |  |  |  |  |  |
|  |  |  |  |  |  |  |  |  |
| 8 |  | **Only including patients with SAPS3 in the 1st quartile** | | | | | | |
|  |  | Age |  | 91.57 |  | 39.04 |  | 214.78 |
|  |  | ICU LoS |  | 0.93 |  | 0.81 |  | 1.07 |
|  |  | Hospital LoS |  | 1.28 |  | 1.05 |  | 1.56 |
|  |  | CCI score |  | 1.07 |  | 0.90 |  | 1.26 |
|  |  | SAPS3 Box 2+3 |  | 1.02 |  | 0.88 |  | 1.19 |
|  |  | RRT - Yes |  | 0.00 |  | 0.00 |  | 1.4E9 |
|  |  | Ventilator - Yes |  | 0.74 |  | 0.58 |  | 0.93 |
|  |  | Sex - Female |  | 1.04 |  | 0.90 |  | 1.21 |
|  |  | Sepsis - Yes |  | 0.98 |  | 0.50 |  | 1.89 |
|  |  |  |  |  |  |  |  |  |
|  |  |  |  |  |  |  |  |  |
| CCI: revised Charlson Comorbidity Index  CI: Confidence Interval  HR: Hazard Ratio | | | | |  |  |  |  |
| ICU: Intensive Care Unit | | |  |  |  |  |  |  |
| LoS: Length of Stay | | |  |  |  |  |  |  |
| RRT: Renal Replacemet Therapy | | | |  |  |  |  |  |
| SAPS3: Simplifyed Acute Physiology Score 3 | | | | |  |  |  |  |
|  |  |  |  |  |  |  |  |  |
